# Multidimensional Health Profiles in Hospitalized Patients with Mental Disorders: A Cluster Analysis of Cardiovascular Risk, Executive Function, and Emotional Burden

**DOI:** 10.3390/healthcare14070868

**Published:** 2026-03-28

**Authors:** Julia Andrea Arias-Diaz, Brayan Patiño-Palma, Carlos Alberto Hurtado-González, Claudia Marcela Cruz-Delgado, Juan Felipe Ayala-Rico, Florencio Arias-Coronel

**Affiliations:** 1 Faculty of Health and Sports Sciences, Fundación Universitaria del Área Andina, Pereira 660001, Colombia; jarias77@areandina.edu.co (J.A.A.-D.); bpatino3@areandina.edu.co (B.P.-P.); ccruz45@areandina.edu.co (C.M.C.-D.); 2 Faculty of Psychology, Universidad Cooperativa de Colombia, Cali 760036, Colombia; carlos.hurtadog@campusucc.edu.co (C.A.H.-G.); juanf.ayalar@gmail.com (J.F.A.-R.); 3 Faculty of Health, Universidad Santiago de Cali, Cali 760035, Colombia

**Keywords:** mental disorders, cardiovascular risk, executive function, cognitive impairment, cluster analysis, psychiatric hospitalization

## Abstract

Background: Mental disorders are often associated with a high burden of physical comorbidities, particularly cardiovascular risk factors, which contribute to functional impairment. However, limited evidence exists regarding the multidimensional interaction between cardiovascular risk, cognitive function, and emotional symptoms in hospitalized psychiatric patients. Objective: This study aimed to examine the relationship between cardiovascular risk and cognitive impairment and to characterize multidimensional health profiles through cluster analysis in hospitalized patients with mental disorders. Methods: An observational, cross-sectional study was conducted in Colombia (N = 50). Cardiovascular risk (Framingham score and anthropometry), cognitive performance (MoCA, DRS-2, FAB), emotional symptoms (BAI, BDI-II, Yesavage, GHQ-28), and functional status (Barthel, Lawton–Brody) were assessed. Hierarchical cluster analysis and Spearman correlations (rho) were used for data analysis. Results: Two distinct clusters were identified. Differentiation was primarily driven by emotional symptom severity and executive dysfunction rather than traditional cardiovascular risk factors. Cluster 2 represented a high-vulnerability profile, characterized by severe anxiety, depression, and significant cognitive impairment (MoCA: 10.3 +/− 5.1). Robust positive correlations were found between BDI-II and Yesavage (rho = 0.91; *p* < 0.001) and between MoCA and FAB (rho = 0.81; *p* < 0.001). Negative correlations confirmed that age (rho = −0.45) and depressive symptoms (rho = −0.32) significantly impacted functional independence (Lawton–Brody). Conclusions: In hospitalized psychiatric patients, multidimensional health profiles are defined by emotional burden and executive dysfunction rather than traditional cardiovascular risk factors. Identifying the high-risk Cluster 2 underscores the need for integrated, multidisciplinary care models that simultaneously address mental health, cognition, and functionality to improve clinical outcomes.

## 1. Introduction

Mental disorders represent a significant burden on global health, being one of the leading causes of disability and reduced quality of life [1]. Traditionally, mental health care has focused on primary psychiatric symptoms; however, growing evidence highlights the high prevalence of medical comorbidities, particularly cardiovascular diseases (CVD), in this population [2]. Patients with mental disorders have a reduced life expectancy, largely attributed to these physical conditions rather than the psychiatric disorders themselves [3]. This intersection between mental and physical health calls for an integrated approach to assessment and treatment.

International studies have established a bidirectional relationship between cardiovascular risk factors (CVRF) and the deterioration of mental and cognitive health. Cohort studies have shown that conditions such as hypertension, diabetes, and obesity not only increase the risk of CVD but are also associated with a higher risk of developing cognitive impairment and mood disorders [4,5]. For instance, metabolic syndrome has consistently been linked with poorer performance on executive function and memory tests, as well as a higher incidence of depression, suggesting shared pathophysiological mechanisms such as systemic inflammation and oxidative stress [6].

This issue has also been documented in the Latin American and Colombian context. Regional studies report a high prevalence of cardiovascular risk factors in patients with psychiatric diagnoses, aggravated by factors such as sedentary lifestyle, inadequate diets, and barriers to accessing integrated health services [7,8]. In Colombia, studies on specific clinical populations have found a significant burden of cardiometabolic comorbidities in patients with severe mental disorders, highlighting the critical need to implement screening and integrated management strategies [9].

Despite this evidence, there remains a gap in the characterization of multidimensional profiles that capture the complex interaction between cardiovascular risk, cognitive functioning, emotional status, and functional capacity in hospitalized patients with mental disorders. Most studies focus on isolated dimensions, and few employ analytical techniques, such as cluster analysis, to identify homogeneous subgroups with specific clinical needs within this heterogeneous population [10]. This approach would allow for better risk stratification and the personalization of interventions.

Therefore, the aim of this study is to determine the relationship between cardiovascular risk and cognitive impairment and to characterize multidimensional profiles through cluster analysis in a sample of hospitalized patients in a mental health institution in Colombia. By identifying subgroups of patients with different combinations of cardiovascular risk, cognitive impairment, emotional symptoms, and functionality levels, we seek to provide evidence for the design of integrated and multidisciplinary care programs.

## 2. Materials and Methods

### 2.1. Study Design and Type

An observational, analytical, and cross-sectional study was conducted, following the STROBE (Strengthening the Reporting of Observational Studies in Epidemiology) guidelines to ensure reporting transparency. The study employed a multidimensional phenotyping approach through cluster analysis to explore the associations between emotional burden, cognitive function, and cardiovascular risk. The research took place at the inpatient unit of the Hospital Mental Universitario de Risaralda (HOMERIS), a regional referral institution for specialized mental health care in Colombia. Data collection was carried out between August and December 2022, during which patients who met the eligibility criteria were consecutively included

### 2.2. Population and Sample

**Figure 1:**
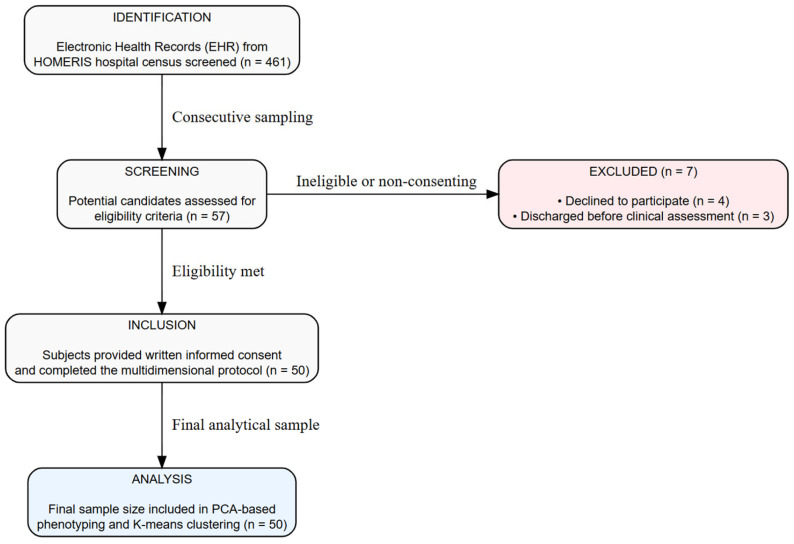
Participant Flow Diagram.

The source population consisted of adult patients hospitalized at the Hospital Mental Universitario de Risaralda during the study period. A non-probabilistic consecutive sampling strategy was employed, using the institutional inpatient census as the sampling frame. A total of 461 medical records were screened; of these, 57 patients initially met the eligibility criteria, and 50 ultimately agreed to participate and provided written informed consent. The remaining seven patients either declined participation or were discharged on the same day as the evaluation. A participant flow diagram is provided in Figure 1 to detail this recruitment process.

Inclusion criteria comprised: age between 46 and 76 years; a minimum of six years of formal education; and a documented medical diagnosis of cardiovascular disease (CVD) or the presence of one or more cardiovascular risk factors (CVRF), such as hypertension, diabetes, or dyslipidemia. This inclusion strategy resulted in a cohort enriched for cardiovascular vulnerability, ensuring a baseline physical risk across all participants. Additionally, the absence of dementia was confirmed through a standardized neurocognitive assessment. Exclusion criteria included severe neuropsychological impairments that interfered with the adequate administration of the assessments, as well as a history of cardiac surgery, due to its potential impact on functional and cognitive performance.

### 2.3. Procedure

Data collection was conducted in a single intrahospital session per participant during daytime hours, in a cubicle designated for clinical assessment with adequate privacy, lighting, and control of environmental distractors. Prior to the evaluation, each participant received a detailed explanation of the study objectives, procedures, potential risks, and benefits, after which written informed consent was obtained.

Assessments were conducted by a standardized team comprising one physiotherapist and one psychologist, both specialists in clinical research. This interdisciplinary duo underwent rigorous prior training in instrument administration and inter-rater reliability procedures to ensure diagnostic consistency. To mitigate systematic measurement bias and potential fatigue effects, all evaluations followed a strictly predetermined execution sequence across the entire study population.

Initially, sociodemographic and clinical variables were obtained through a systematic review of institutional medical records, including age, sex, relevant medical history, smoking status, and diagnosis of diabetes mellitus. Subsequently, anthropometric measurements were recorded in accordance with World Health Organization guidelines, including body weight, height for body mass index (BMI) calculation, and waist and hip circumferences for waist-to-hip ratio estimation [11].

Based on these variables and the clinical information recorded, global cardiovascular risk was estimated using the Framingham Risk Score (FRS), following the model proposed for use in primary care settings [12]. This allowed for a baseline characterization of the cohort’s physical vulnerability profile.

Cognitive and executive screening was performed using widely used and validated instruments: the Montreal Cognitive Assessment (MoCA) for global cognitive assessment [13], the Dementia Rating Scale–2 (DRS-2) for global cognitive performance [14], and the Frontal Assessment Battery (FAB) for executive function [15].

Emotional status was assessed through a multidimensional battery including the Beck Anxiety Inventory (BAI) for anxiety [16], the Beck Depression Inventory–II (BDI-II) for depressive symptoms [17], the Geriatric Depression Scale by Yesavage [18], and the General Health Questionnaire–28 (GHQ-28) for general psychological distress [19].

Functional status was evaluated using the Barthel Index for activities of daily living [20] and the Lawton and Brody scale for instrumental activities of daily living [21]. Lifestyle and recent physical activity were assessed using the Simple Physical Activity Questionnaire (SIMPAQ), a tool validated for psychiatric clinical settings, following official administration and scoring instructions [22].

To ensure measurement validity and reliability, all instruments were administered face-to-face, with literal reading of items when necessary, use of standardized materials, and immediate recording of scores. Blinded cross-checks between evaluators were performed before closing each session, and calibration sessions were conducted prior to study initiation to minimize inter-rater variability.

### 2.4. Statistical Analysis

Data were analyzed using R software (v4.5.1). Continuous variables were described using means and standard deviations (SD), while categorical variables were summarized as absolute frequencies and proportions. To address the redundancy among the five emotional scales (an observation raised during the peer-review process), a Principal Component Analysis (PCA) was performed to derive a synthetic “Emotional Burden Index” (PC1), which accounted for 83% of the shared variance among the instruments [16,17,18,19]. Multidimensional health profiles were identified using K-means clustering (*k* = 2 with 50 random starts to ensure centroid stability). The optimal number of clusters was determined using the elbow method and the average silhouette coefficient. The clustering algorithm was fed with a standardized matrix comprising age, BMI, systolic blood pressure, cognitive scores (MoCA, DRS-2, FAB), functional status, and the PCA-derived Emotional Burden Index. Given the non-normal distribution of several variables, between-cluster comparisons were conducted using the Wilcoxon rank-sum test.

Beyond *p*-values, effect sizes were quantified using Rosenthal’s *r* coefficient (Small < 0.3; Moderate = 0.3–0.5; Large > 0.5) to evaluate the clinical magnitude of the differences despite the sample size (N = 50). *p*-values were adjusted for multiple comparisons using the Benjamini–Hochberg (BH) method to control the false discovery rate. Associations between clinical, cognitive, and functional variables were estimated using Spearman’s rank correlation coefficient (rho). Furthermore, to control for age as a significant confounding factor in cognitive performance, partial Spearman correlations were conducted between emotional burden and global cognition (MoCA).

### 2.5. Ethical Considerations

The study was conducted in accordance with the ethical principles of the Declaration of Helsinki and current Colombian regulations (Resolution 8430 of 1993 issued by the Ministry of Health and Law 1581 of 2012 on personal data protection). The protocol was reviewed and approved by the Institutional Ethics and Bioethics Committee for Research (CEBIS) of Fundación Universitaria del Área Andina (Approval Act No. 07, 11 May 2022). All participants provided written informed consent prior to inclusion in the study.

## 3. Results

The general characteristics of the 50 participants are summarized in Table 1. The cohort showed a mean age of 57.9 ± 10.1 years and a female predominance (64%). Metabolic and cardiovascular risk factors were highly prevalent, including current smoking (56%) and diabetes mellitus (34%), alongside a pervasive sedentary lifestyle (86%). Functional assessment via the Lawton & Brody scale revealed that 60% of subjects exhibited deficits in instrumental activities of daily living.

The cluster analysis identified two distinct groups with contrasting clinical profiles. Cluster 2 (*n* = 23) was significantly older (61.8 ± 10.3 years; *p* = 0.020) and presented a higher metabolic risk profile compared to Cluster 1 (*n* = 27), evidenced by a higher BMI (28.7 ± 7.2 vs. 23.9 ± 5.4; *p* = 0.027) and an elevated Waist-to-Hip Ratio (0.94 ± 0.06 vs. 0.88 ± 0.08; *p* = 0.019). Systolic blood pressure and diabetes prevalence did not differ significantly between groups (*p* > 0.05).

Regarding neurocognitive performance, Cluster 2 exhibited significantly lower scores across all measures: MoCA (10.3 ± 3.4), DRS-2 (93.8 ± 17.7), and FAB (7.0 ± 3.7). Conversely, Cluster 1 showed higher preservation in global cognition (MoCA: 21.4 ± 4.6) and executive function (FAB: 14.6 ± 2.5) (Table 1). These disparities were supported by large effect sizes (r > 0.70), confirming a robust statistical separation between the clusters. Although Cluster 2 showed a higher frequency of instrumental functional deficit (73.9%) compared to Cluster 1 (48.1%), this difference did not reach statistical significance (*p* = 0.086).

To determine the optimal number of clusters, two complementary mathematical criteria were employed: the average silhouette coefficient and the elbow method (Within-Cluster Sum of Squares, WSS).

**Figure 2:**
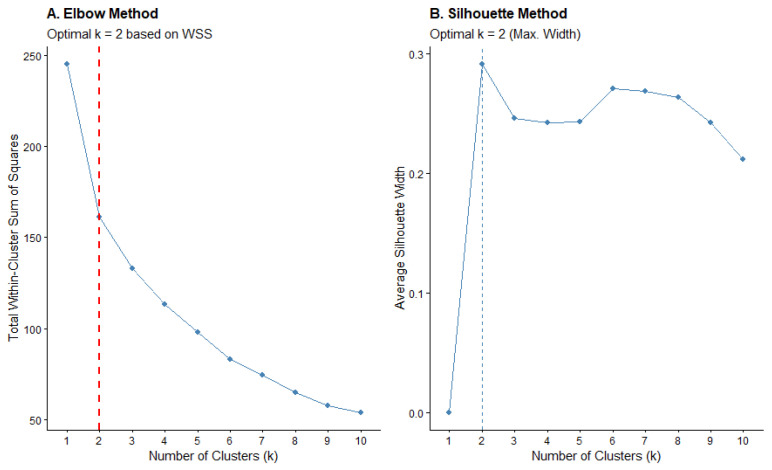
Determination of the optimal number of clusters: silhouette and elbow method.

The silhouette analysis (Figure 2B) jointly evaluated intra-cluster cohesion and inter-cluster separation; the maximum average silhouette width was achieved at k = 2 (represented by the dashed line), indicating a robust and well-differentiated partition. This was corroborated by the elbow method (Figure 2A), which displayed a clear inflection point at k = 2, where further increases in k did not substantially improve data compactness. Based on this convergence, a non-hierarchical K-means clustering algorithm was adopted for the final segmentation of the 50 participants into two distinct phenotypes.

**Figure 3:**
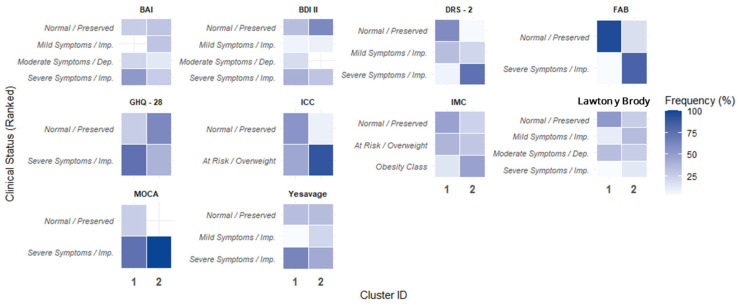
Heatmap of categories by cluster.

Based on the hierarchical cluster analysis with k = 2, two clearly differentiated groups were identified. Their overall characterization is presented in Figure 3 using a heatmap that summarizes the percentage distribution of categories by variable. Cluster 2 was distinguished by a higher burden of severe emotional disorders (severe anxiety on the BAI and GHQ-28; severe depression on the BDI-II and Yesavage scales), functional impairment (moderate-to-severe dependence according to the Lawton and Brody scale), and marked cognitive alterations in MoCA and DRS-2.

Additionally, this subgroup had a higher prevalence of obesity and sedentary behavior. In contrast, Cluster 1 comprised a higher proportion of participants with results within normal ranges in mental health, cognition, and functional status, exhibiting more preserved profiles across the evaluated domains. The contrast between clusters revealed statistically significant differences across multiple dimensions of the emotional domain, supporting the clinical validity of the segmentation. Specifically, comparative analyses showed adjusted *p*-values < 0.01 for anxiety (BAI), depression (BDI-II and Yesavage), and general psychological distress (GHQ-28), with greater impairment in Cluster 2. This pattern is consistent with the categorical distribution shown in Figure 3, where Cluster 2 concentrates cases with moderate-to-severe symptoms and impaired quality of life, whereas Cluster 1 remains predominantly within normal categories.

Regarding the cognitive and executive domains, classifications based on the FAB, MoCA, and DRS-2 followed a complementary pattern. Cluster 2 comprised the majority of participants classified with cognitive impairment (FAB) and severe impairment (MoCA and DRS-2), with statistically significant differences (adjusted *p*-values < 0.01). In the clinical domain, while age and cardiovascular risk factors showed significant trends, anthropometric variables such as BMI classification and HEARTS risk strata did not differ as sharply, indicating that group differentiation in this population is primarily driven by the psycho-emotional burden and the cognitive–executive profile.

**Figure 4:**
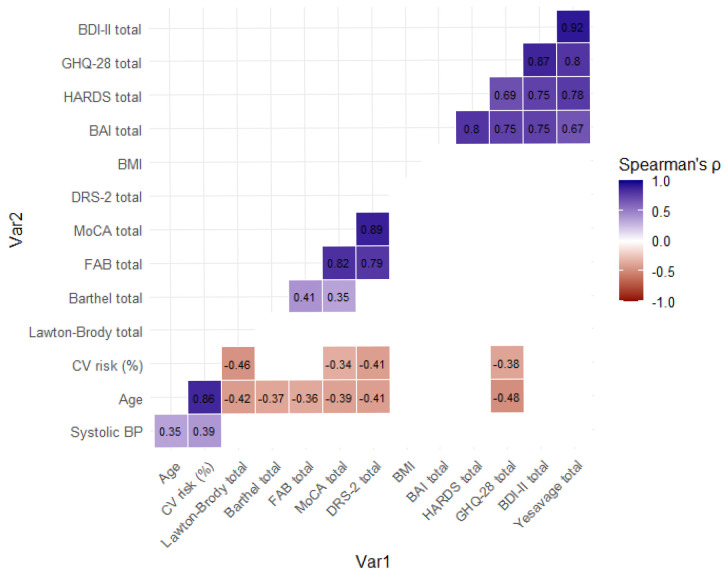
Heatmap of significant Spearman correlations among total variables.

To evaluate the clinical and functional associations across the entire sample, a Spearman correlation analysis was performed, revealing a network of significant relationships between cognitive, emotional, and functional domains (Figure 4).

A high degree of convergent validity was observed among cognitive instruments: MoCA scores correlated strongly with both DRS-2 (*rho* = 0.89) and FAB (*rho* = 0.82), while DRS-2 also exhibited a robust association with FAB (*rho* = 0.79). Mental health indicators showed similarly high internal consistency; the strongest correlation within the emotional domain was identified between the BDI-II and Yesavage scales (*rho* = 0.92), followed by the association between BDI-II and GHQ-28 (*rho* = 0.87). Anxiety (BAI) was consistently associated with psychological distress (HARDS: rho = 0.80), general psychological health (GHQ-28: *rho* = 0.75), and depressive symptoms (BDI-II: *rho* = 0.75).

Furthermore, the correlational network delineated significant inverse associations between demographic factors and functional outcomes. Age was negatively associated with functional independence on the Lawton–Brody scale (*rho* = −0.42), global cognition (DRS-2: *rho* = −0.41; MoCA: *rho* = −0.39), and executive performance on the FAB (*rho* = −0.36). Notably, age showed its strongest positive correlation with cardiovascular risk (*rho* = 0.86). 

Regarding the impact of cardiovascular health, CV risk was negatively related to functional autonomy (Lawton-Brody: *rho* = −0.46) and cognitive status (DRS-2: *rho* = −0.41; MoCA: *rho* = −0.34). Finally, the analysis revealed that higher systolic blood pressure was associated with both age (*rho* = 0.35) and cardiovascular risk (*rho* = 0.39), while the Barthel Index showed moderate positive correlations with executive and global cognitive function (FAB: *rho* = 0.41; MoCA: *rho* = 0.35).

## 4. Discussion

A crucial finding of the present analysis was that the primary differentiation between the identified profiles was not driven by traditional cardiovascular risk factors, such as age, body mass index, or smoking, which showed no significant differences, but rather by a marked burden of emotional symptoms (anxiety and depression) and, secondarily, by executive function impairment. This finding aligns with emerging literature that recognizes internalizing disorders as a central axis in the pathophysiology and prognosis of patients with medical comorbidities [23,24]. In this context, a recent study in patients with structural cardiovascular disease identified psychological distress, beyond traditional risk factors, as an independent predictor of adverse clinical outcomes [25]. In the present study, this configuration defines the existence of a subgroup with high psycho-emotional vulnerability (Cluster 2) that, regardless of baseline cardiovascular risk profile, exhibits a constellation of symptoms, supported by the strong correlation between depressive and anxiety symptoms (rho = 0.91 between BDI-II and Yesavage), that exacerbate disability and cognitive deterioration. These results reinforce the predominance of mental health as a key differentiating axis even in populations with a high prevalence of physical comorbidity, requiring prioritized and integrated clinical care [24,26].

Cluster 2, characterized by high levels of anxiety, depression, and psychological distress, together with executive dysfunction, represents a subgroup with high psycho-emotional vulnerability. This profile is consistent with previous studies identifying psychological distress as an independent predictor of adverse clinical events and mortality, even surpassing conventional cardiovascular risk factors [23,26]. Our findings extend this evidence to hospitalized patients with mental disorders, suggesting that emotional burden acts as a key factor exacerbating disability and cognitive–functional impairment [27]. This is statistically supported by the robust correlations found between emotional distress and cognitive-executive performance, such as the association between depressive symptoms (Yesavage) and functional autonomy (Lawton–Brody: rho = −0.32; *p* < 0.05), as well as the overlap between anxiety (BAI) and multiple distress indicators (rho > 0.70).

The negative correlation observed between cardiovascular risk, particularly central adiposity (ICC) and systolic blood pressure, and performance on cognitive and functional measures further supports the presence of a shared pathophysiological interplay. As shown in the correlational network, higher systolic blood pressure was associated with poorer functional performance on the Barthel Index (rho = −0.47; *p* < 0.05), while age and cardiovascular risk indicators showed a clear inverse relationship with executive function (FAB: rho = −0.47). Several mechanisms have been proposed to explain this relationship, including low-grade systemic inflammation, oxidative stress, and endothelial dysfunction, which may constitute a common biological substrate linking cardiovascular disease, cognitive impairment, and mood disorders in the vulnerable Cluster 2 [28,29]. These findings suggest that in this cohort, the physiological burden of vascular risk directly compromises the neural networks subserving executive control and functional independence.

Despite the high prevalence of sedentary behavior and cardiometabolic risk in the overall sample, these variables did not define cluster segmentation as independent factors. This is consistent with the lack of significant linear correlations between BMI and most cognitive or emotional indicators in the overall sample. This suggests that, although they represent common health issues in this population, their impact on the configuration of more specific clinical profiles may be mediated or amplified by the patient’s emotional and cognitive status [30]. In this regard, executive function emerged as a relevant differentiating factor, supported by the strong association between global and executive measures (MoCA and FAB: rho = 0.81; *p* < 0.001). These findings support the hypothesis that impairment in higher-order cognitive domains, such as planning, cognitive flexibility, and inhibitory control, constitutes a critical link between emotional disturbance and the capacity for self-regulation of physical health and functional performance in the high-risk Cluster 2 [31].

### 4.1. Limitations

Several limitations should be considered. Firstly, the cross-sectional design precludes causal inferences regarding the observed relationships between emotional distress and cognitive decline. Secondly, although the sample size ($n = 50$) was adequate for the cluster analysis performed, it limits the generalizability of the findings and the detection of differences in some secondary variables. Finally, the study was conducted in a single hospital setting, highlighting the need for future research in other clinical contexts and with larger samples to confirm these results and further explore the longitudinal impact of the metabolic-cognitive interplay identified in Cluster 2.

### 4.2. Clinical Implications

From a clinical perspective, the identification of a subgroup with high psycho-emotional and cognitive vulnerability (Cluster 2) underscores the need to implement systematic assessments of emotional status and executive function in psychiatric inpatient services [32]. Our findings, supported by the strong correlations between anxiety, depression (rho = 0.91), and executive impairment (FAB: rho = −0.47), demonstrate that the isolated detection of cardiovascular risk factors is insufficient for comprehensive risk stratification. Integrated and multidisciplinary care models, combining psychiatric, psychological, cognitive, and cardiometabolic approaches, are required to design personalized interventions aimed at improving functioning and quality of life in this patient population [33,34]. Specifically, for the high-risk profile identified, interventions should prioritize cognitive-executive rehabilitation and emotional regulation as a means to improve self-care and reduce the long-term burden of physical comorbidity

## 5. Conclusions

In conclusion, the present study demonstrates that, in hospitalized patients with mental disorders, multidimensional health profiles are defined primarily by the burden of emotional symptoms and executive function impairment rather than by traditional cardiovascular risk factors. Through cluster analysis, a subgroup with high psycho-emotional vulnerability (Cluster 2) was identified, characterized by significantly higher levels of anxiety, depression, psychological distress, and executive dysfunction (MoCA: 10.3), which are associated with poorer functioning and reduced quality of life. These findings, supported by robust correlations between emotional distress and functional independence (rho = −0.32 with Lawton–Brody) and among cognitive-executive measures (rho = 0.81 between MoCA and FAB), highlight the need to move beyond evaluation models focused exclusively on cardiometabolic risk. It is essential to implement integrated, multidisciplinary care approaches that systematically incorporate emotional, cognitive, and functional assessments. Early identification of these high-risk profiles may guide more personalized clinical strategies, optimize therapeutic planning, and contribute to improving functional outcomes and quality of life in individuals with mental illness.

## Figures and Tables

**Table 1:** Multidimensional baseline characteristics and clinical phenotypes of the study population. (N = 50).

Variable	Total (N = 50)	Cluster 1 (n = 27)	Cluster 2 (n = 23)	*p*-Value	Effect Size (*r*)
**Sociodemographic Characteristics**					
**Age (years)**	57.9 (10.1)	54.7 (9.0)	61.8 (10.3)	0.020	0.328
**Sex, Female n (%)**	32 (64%)	14 (51.9%)	18 (78.3%)	0.077	-
**Metabolic & Cardiovascular Risk**					
**BMI (kg/m^2^)**	26.1 (6.7)	23.9 (5.4)	28.7 (7.2)	0.027	0.313
**Waist-to-Hip Ratio**	0.91 (0.07)	0.88 (0.08)	0.94 (0.06)	0.019	0.330
**Systolic BP (mmHg)**	111.6 (18.9)	112.5 (13.9)	110.4 (23.8)	0.534	0.088
**Diabetes Mellitus n (%)**	17 (34%)	8 (29.6%)	9 (39.1%)	0.557	-
**Functional & Neurocognitive Performance**					
**Lawton & Brody n (%)**	30 (60%)	13 (48.1%)	17 (73.9%)	0.086	-
**MoCA (Total score)**	16.3 (6.9)	21.4 (4.6)	10.3 (3.4)	<0.001	0.834
**DRS-2 (Total score)**	110.3 (21.1)	124.4 (11.3)	93.8 (17.7)	<0.001	0.730
**FAB (Total score)**	11.1 (4.9)	14.6 (2.5)	7.0 (3.7)	<0.001	0.781
**Lifestyle & Habits**					
**Sedentary n (%)**	43 (86%)	22 (81.5%)	21 (91.3%)	0.43	-
**Smoking Status n (%)**	28 (56%)	18 (66.7%)	10 (43.5%)	0.153	-

Note: Continuous variables as Mean (SD); Categorical as n (%).

## Data Availability

The datasets generated and analyzed during the current study are not publicly available due to institutional regulations and ethical restrictions related to patient confidentiality and data protection laws in Colombia (Law 1581 of 2012). However, anonymized data are available from the corresponding author upon reasonable request and with permission from the Ethics Committee of Fundación Universitaria del Área Andina (CEBIS).
